# Unveiling Local Electronic Structure of Lanthanide‐Doped Cs_2_NaInCl_6_ Double Perovskites for Realizing Efficient Near‐Infrared Luminescence

**DOI:** 10.1002/advs.202203735

**Published:** 2022-09-30

**Authors:** Siyuan Han, Datao Tu, Zhi Xie, Yunqin Zhang, Jiayao Li, Yifan Pei, Jin Xu, Zhongliang Gong, Xueyuan Chen

**Affiliations:** ^1^ CAS Key Laboratory of Design and Assembly of Functional Nanostructures Fujian Key Laboratory of Nanomaterials and State Key Laboratory of Structural Chemistry Fujian Institute of Research on the Structure of Matter Chinese Academy of Sciences Fuzhou Fujian 350002 China; ^2^ University of Chinese Academy of Sciences Beijing 100049 China; ^3^ Fujian Science & Technology Innovation Laboratory for Optoelectronic Information of China Fuzhou Fujian 350108 China; ^4^ College of Mechanical and Electronic Engineering Fujian Agriculture and Forestry University Fuzhou Fujian 350002 China

**Keywords:** charge transfer, double perovskites, lanthanide ions, local electronic structure, near‐infrared luminescence

## Abstract

Lanthanide ion (Ln^3+^)‐doped halide double perovskites (DPs) have evoked tremendous interest due to their unique optical properties. However, Ln^3+^ ions in these DPs still suffer from weak emissions due to their parity‐forbidden 4f–4f electronic transitions. Herein, the local electronic structure of Ln^3+^‐doped Cs_2_NaInCl_6_ DPs is unveiled. Benefiting from the localized electrons of [YbCl_6_]^3−^ octahedron in Cs_2_NaInCl_6_ DPs, an efficient strategy of Cl^−^‐Yb^3+^ charge transfer sensitization is proposed to obtain intense near‐infrared (NIR) luminescence of Ln^3+^. NIR photoluminescence (PL) quantum yield (QY) up to 39.4% of Yb^3+^ in Cs_2_NaInCl_6_ is achieved, which is more than three orders of magnitude higher than that (0.1%) in the well‐established Cs_2_AgInCl_6_ via conventional self‐trapped excitons sensitization. Density functional theory calculation and Bader charge analysis indicate that the [YbCl_6_]^3−^ octahedron is strongly localized in Cs_2_NaInCl_6_:Yb^3+^, which facilitates the Cl^−^‐Yb^3+^ charge transfer process. The Cl^−^‐Yb^3+^ charge transfer sensitization mechanism in Cs_2_NaInCl_6_:Yb^3+^ is further verified by temperature‐dependent steady‐state and transient PL spectra. Furthermore, efficient NIR emission of Er^3+^ with the NIR PLQY of 7.9% via the Cl^−^‐Yb^3+^ charge transfer sensitization is realized. These findings provide fundamental insights into the optical manipulation of Ln^3+^‐doped halide DPs, thus laying a foundation for the future design of efficient NIR‐emitting DPs.

## Graphical abstract

The authors achieve efficient near‐infrared (NIR) luminescence in lanthanide‐doped Cs_2_NaInCl_6_, with a photoluminescence quantum yield of Yb^3+^ up to 39.4% via Cl^−^‐Yb^3+^ charge transfer sensitization. Temperature‐dependent spectroscopic and theory calculations reveal the characteristic local electron structure of t [YbCl_6_]^3−^ octahedron in Yb^3+^‐doped Cs_2_NaInCl_6_, which boosts the NIR emissions of Yb^3+^ and Er^3+^ through Cl^−^‐Yb^3+^ charge transfer sensitization.

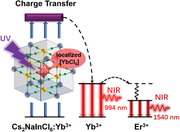

## 1 Introduction

Lead‐free double perovskites (DPs) with A_2_B^I^B^III^X_6_ stoichiometry have attracted much attention in recent years due to their good stability, low toxicity, and diversity of composition.^[^ ^1^ ^]^ These DPs are characterized by a 3D structure composed of alternating [B^+^X_6_] and [B^3+^X_6_] corner‐sharing octahedron with A^+^ ions occupying the voids. Several combinations for A_2_B^I^B^III^X_6_ DPs have been reported, wherein B^+^ can be Ag^+^, Na^+^, Li^+^, K^+^ and B^3+^ can be In^3+^, Sb^3+^, Bi^3+^, Tl^3+^, etc.^[^ ^2^ ^]^ Despite the attractive photophysical properties of these A_2_B^I^B^III^X_6_ DPs, their studies were mainly restricted to the visible spectral region. Hitherto, it is quite challenging to realize efficient near‐infrared (NIR) luminescence in these DPs.

To this regard, lanthanide ions (e.g., Yb^3+^, Er^3+^, Tm^3+^) with rich electronic energy levels were proposed for tailoring the optical performances of DPs toward the NIR regions. Among various A_2_B^I^B^III^X_6_ DPs, Cs_2_Na(Ag)InCl_6_ DPs have been widely reported as one of the excellent hosts for Ln^3+^ doping owing to the direct bandgap character and high chemical stability. It was reported that NIR emission from Yb^3+^ can be produced in Cs_2_AgInCl_6_ DPs via the sensitization of self‐trapped exciton (STE).^[^ ^3^ ^]^ For example, Kim et al. doped Yb^3+^/Er^3+^ into Cs_2_AgInCl_6_ nanocrystals, which exhibited characteristic NIR emissions of Yb^3+^ and Er^3+^ peaking at 996 and 1537 nm, respectively.^[^ ^4^ ^]^ However, the NIR photoluminescence (PL) quantum yield (QY) of these Ln^3+^‐doped Cs_2_AgInCl_6_ DPs remains low (<5%). Thus, substantial efforts have been made to overcome such obstacles and to enhance the NIR luminescence of Ln^3+^‐doped DPs. Typically, sensitizer (e.g., Bi^3+^) co‐doping or Na^+^/Ag^+^ alloying strategies have to be adopted. Nag et al. boosted the NIR emission of Ln^3+^ in Cs_2_AgInCl_6_ through co‐doping with Bi^3+^, which introduced a new optical absorption channel to sensitize the Ln^3+^ dopants of Yb^3+^ and Er^3+^.^[^ ^5^ ^]^ Lin et al. synthesized Bi^3+^/Yb^3+^ co‐doped Cs_2_Na_0.6_Ag_0.4_InCl_6_ DPs, in which Na^+^/Ag^+^ alloying broke the local site symmetry of Cs_2_AgInCl_6_ to enhance the NIR emission of Bi^3+^‐sensitized Yb^3+^.^[^ ^6^ ^]^ Because the optical transitions of Ln^3+^ are sensitive to the local coordination, the PLQY of Ln^3+^ ions in these lead‐free DPs strongly depends on the crystal structure around Ln^3+^. Unfortunately, the local electronic structure of Ln^3+^‐doped Cs_2_NaInCl_6_ and Cs_2_AgInCl_6_ DPs remains essentially untouched yet. To circumvent the complicated energy transfer procedures and difficulty of composition regulation, an unambiguous local structural analysis is a prerequisite to optimizing their optical performance for further applications.

Herein, we propose a facile strategy to boost the NIR luminescence of Ln^3+^ (Yb^3+^ and Er^3+^) in Cs_2_NaInCl_6_ DPs. Through a theoretical survey of the local electronic structure based on density functional theory (DFT) and Bader charge analysis calculations, we revealed that the characteristic local electronic structure of [YbCl_6_]^3−^ octahedron in Cs_2_NaInCl_6_ DPs can greatly promote the Cl^−^‐Yb^3+^ charge transfer process. Benefiting from the Cl^−^‐Yb^3+^ charge transfer sensitization, intense NIR emission of Yb^3+^ in Cs_2_NaInCl_6_ DPs was achieved, with an intensity 142.2 times higher than the well‐established Cs_2_AgInCl_6_:Yb^3+^ counterparts. Temperature‐dependent PL spectroscopic measurements confirmed the efficient energy transfer path from Cl^−^‐Yb^3+^ charge transfer band (CTB) to Yb^3+^.^[^ ^7^ ^]^ Furthermore, we also achieved intense NIR emission of Er^3+^ in Cs_2_NaInCl_6_:Yb^3+^/Er^3+^ through Cl^−^‐Yb^3+^ charge transfer sensitization, the integrated intensity of which was 1510.2 times higher than that of Cs_2_NaInCl_6_:Er^3+^ counterparts, respectively.

## 2 Results and Discussion

**Figure 1:**
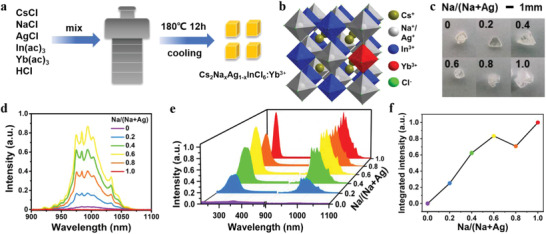
a) Schematic diagram of the synthesis of Cs_2_Na*_x_* Ag_1−_ *_x_* InCl_6_ and Cs_2_Na*_x_* Ag_1−_ *_x_* InCl_6_:Yb^3+^ crystals with different Na/(Na+Ag) ratios. b) Crystal structure of Cs_2_Na*_x_* Ag_1−_ *_x_* InCl_6_:Yb^3+^. c) Photographs of Cs_2_Na*_x_* Ag_1−_ *_x_* InCl_6_:Yb^3+^ crystals. d) PL emission spectra of Cs_2_Na*_x_* Ag_1−_ *_x_* InCl_6_:Yb^3+^ crystals excited by 365 nm. e) PL excitation (left) and emission (right) spectra of Cs_2_Na*_x_* Ag_1−_ *_x_* InCl_6_:Yb^3+^ crystals. f) Integrated emission intensity of Yb^3+^ in Cs_2_Na*_x_* Ag_1−_ *_x_* InCl_6_:Yb^3+^ crystals with different Na/(Na+Ag) ratios.

Cs_2_Na*_x_* Ag_1−_ *_x_* InCl_6_ and Cs_2_Na*_x_* Ag_1−_ *_x_* InCl_6_:Yb^3+^ crystals with different Na/Ag ratios were synthesized via a hydrothermal method (**Figure** **1**a). X‐ray diffraction (XRD) patterns of the crystals can be well indexed into cubic Cs_2_AgInCl_6_ (ICSD No. 244519) and Cs_2_NaInCl_6_ (ICSD No. 132718) without any impurities (Figure S1, Supporting Information), which indicates that the as‐prepared Cs_2_Na*_x_* Ag_1−_ *_x_* InCl_6_:Yb^3+^ crystals have the typical double perovskite structure with space group of *Fm* $\bar{3}$ *m* (Figure 1b). These crystals were transparent with the size of several millimeters (Figure 1c). The absorption band of Cs_2_Na*_x_* Ag_1−_ *_x_* InCl_6_:Yb^3+^ crystals located in the UV region, and band edges monotonically shifted from 355 to 283 nm as the Na/(Na+Ag) ratio increased from 0 to 1 (Figure S2, Supporting Information).

Upon excitation at 365 nm, NIR emission of Yb^3+^ can be produced in these Cs_2_Na*_x_* Ag_1−_ *_x_* InCl_6_:Yb^3+^ DPs. The optimal NIR emission of Yb^3+^ was obtained when Na/(Na+Ag) ratio was 0.6 as reported previously (Figure 1d).^[^ ^3^, ^8^ ^]^ However, it should be noted that the excitation peaks exhibited an obvious blue shift from 350 to 273 nm and the shape of the peaks became sharper with the Na/(Na+Ag) ratio rising from 0 to 1 (Figure 1e). Upon excitation with the best excitation wavelength of these Cs_2_Na*_x_* Ag_1−_ *_x_* InCl_6_:Yb^3+^ DPs, it was observed that the NIR luminescence intensity of Yb^3+^ markedly increased by 135.6 times as the Na/(Na+Ag) ratio increased from 0 to 1 (Figure 1f). According to the PL decays of Yb^3+^, the lifetime of Yb^3+^ increased from 2.72 to 4.52 ms with increasing the Na/(Na+Ag) ratio from 0 to 1 (Figure S2, Supporting Information). Intriguingly, Cs_2_NaInCl_6_:Yb^3+^ exhibited the highest NIR luminescence intensity and longest PL lifetime of Yb^3+^ among the Cs_2_Na*_x_* Ag_1−_ *_x_* InCl_6_:Yb^3+^ DPs, which had not been reported before.

**Figure 2:**
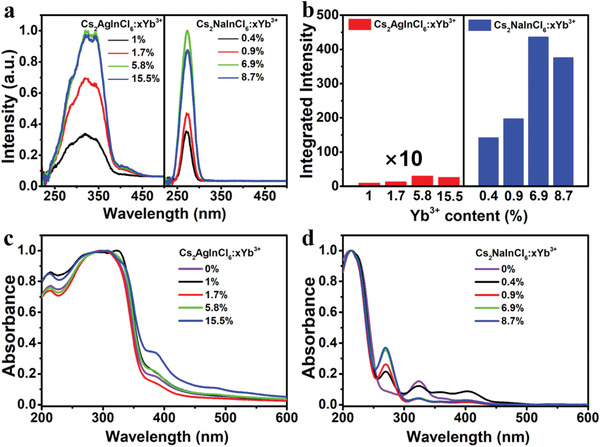
a) PL excitation spectra of Cs_2_AgInCl_6_:Yb^3+^ and Cs_2_NaInCl_6_:Yb^3+^ with different Yb^3+^ concentrations. b) Integrated Yb^3+^ emission intensity of Cs_2_AgInCl_6_:Yb^3+^ and Cs_2_NaInCl_6_:Yb^3+^ with different Yb^3+^ concentrations. c) Diffuse reflectance spectra of Cs_2_AgInCl_6_:Yb^3+^ with different Yb^3+^ concentrations. d) Diffuse reflectance spectra of Cs_2_NaInCl_6_:Yb^3+^ with different Yb^3+^ concentrations.

To explore the NIR luminescence mechanism of Yb^3+^ in Cs_2_AgInCl_6_ and Cs_2_NaInCl_6_, we synthesized Cs_2_AgInCl_6_:Yb^3+^ and Cs_2_NaInCl_6_:Yb^3+^ DPs with different contents of Yb^3+^. XRD patterns confirmed the pure phase of these samples (Figure S3, Supporting Information). X‐ray photoelectron spectra analysis revealed the existence of Yb^3+^ ions in the as‐prepared DPs (Figure S4, Supporting Information). For Cs_2_AgInCl_6_, the feeding concentrations of Yb^3+^ were from 50% to 200%, while the actual Yb^3+^ concentrations in the crystal lattice were identified to be only from 1% to 15.5% based on the inductively coupled plasma atomic emission spectra analysis (Table S1, Supporting Information).^[^ ^3a^ ^]^ By monitoring the Yb^3+^ emission at 994 nm, a broad excitation band (250–400 nm) centered at ≈350 nm was detected (**Figure** **2**a), which was associated with the bandgap absorption of Cs_2_AgInCl_6_. Upon excitation at 365 nm, Cs_2_AgInCl_6_:Yb^3+^ with different Yb^3+^ concentrations exhibited weak NIR PL (Figure S5, Supporting Information and Figure 2b). PL decays revealed decreased PL lifetime from 2.76 to 2.54 ms with the concentration of Yb^3+^ from 1.0% to 15.5% (Figure S5, Supporting Information). Diffuse reflectance spectra of Cs_2_AgInCl_6_:Yb^3+^ exhibited an intense absorption at ≈358 nm (3.47 eV) (Figure 2c), which agrees well with the absorption spectrum of pure Cs_2_AgInCl_6_.^[^ ^2b^ ^]^

For Cs_2_NaInCl_6_, we adopted the same feeding concentrations as those in Cs_2_AgInCl_6_, resulting in also low concentrations of Yb^3+^ from 0.4% to 8.7% into the Cs_2_NaInCl_6_ lattice (Tables S2 and S3, Supporting Information). When monitoring the Yb^3+^ emission of 994 nm, a sharp excitation peak at 273 nm was detected, which was ≈70 nm blue‐shift compared with that of Cs_2_AgInCl_6_:Yb^3+^ (Figure 2a). Meanwhile, the full‐width of half‐maximum (FWHM) of the excitation peak (≈30 nm) was much narrower than that (≈60 nm) of Cs_2_AgInCl_6_:Yb^3+^. Diffuse reflectance spectrum of pure Cs_2_NaInCl_6_ exhibited an ultra‐weak absorption band in the visible region and the bandgap was determined to be 4.45 eV (Figure 2d).^[^ ^2^, ^9^ ^]^ However, a new and sharp absorption peak appeared at ≈273 nm when Yb^3+^ was introduced in Cs_2_NaInCl_6_. With increasing the Yb^3+^ concentration, this absorption peak increased and reached the strongest when the Yb^3+^ concentration was 6.9%. According to the previous report, this sharp excitation peak can be well conformed to the CTB absorption.^[^ ^10^ ^]^ Particularly, upon excitation at 273 nm, the NIR luminescence intensity of Yb^3+^ was observed to be 142.2 times higher than that of the Cs_2_AgInCl_6_:Yb^3+^ counterpart with the optimal doping concentration (Figure 2b). The highest PLQY of Yb^3+^ in Cs_2_NaInCl_6_:Yb^3+^ reaches 39.4%, which is higher than most of the lead‐free halide DPs (Table S4, Supporting Information). Note that the NIR PLQY of Cs_2_AgInCl_6_:Yb^3+^ counterpart was less than 0.1% under otherwise identical conditions. Furthermore, the PL lifetime of Yb^3+^ in Cs_2_NaInCl_6_:Yb^3+^ was determined to decrease from 4.54 to 4.11 ms with the increase of Yb^3+^ concentration from 0.4% to 8.7% (Figure S5, Supporting Information), which was much longer than that in Cs_2_AgInCl_6_:Yb^3+^.

**Figure 3:**
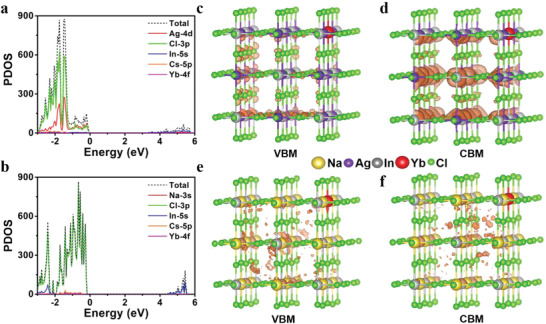
Partial density of states for a) Cs_2_AgInCl_6_:Yb^3+^ and b) Cs_2_NaInCl_6_:Yb^3+^. Orbital distribution profiles of c) VBM and d) CBM in Cs_2_AgInCl_6_:Yb^3+^ (Cs atoms are not displayed). Orbital distribution profiles of e) VBM and f) CBM in Cs_2_NaInCl_6_:Yb^3+^ (Cs atoms are not displayed).

To shed more light on the NIR luminescent mechanism of Yb^3+^, first‐principles calculations based on hybrid DFT were carried out. We replaced the central In^3+^ ion with Yb^3+^ ion in a 2 × 2 × 2 supercell of Cs_2_AgInCl_6_:Yb^3+^ and Cs_2_NaInCl_6_:Yb^3+^ (Figure S8, Supporting Information). The bandgaps of Cs_2_AgInCl_6_:Yb^3+^ and Cs_2_NaInCl_6_:Yb^3+^ were determined to be 3.21 and 4.38 eV, respectively, wherein Yb^3+^ made no contributions to the valence band maximum (VBM) or conduction band minimum (CBM) (**Figure** **3**a,b). The partial density of states analysis and orbital distribution profiles of Cs_2_AgInCl_6_:Yb^3+^ showed that VBM was composed of mixed configuration of Ag 4d and Cl 3p states, and CBM mainly consisted of In 5s states with minor contributions from Ag 4d and Cl 3p states (Figure 3c,d). Such configuration benefited the formation of STE, which resulted from the Jahn–Teller distortion of the connected [AgCl_6_]^5−^‐[InCl_6_]^3−^ octahedron.^[^ ^2b^ ^]^ For Cs_2_NaInCl_6_:Yb^3+^, VBM and CBM were essentially composed of Cl 3p states and In 5s states, respectively, which revealed that the orbitals were distributed over the whole supercell with little spatial overlap (Figure 3e,f). Such poor spatial overlap led to the extremely weak edge‐to‐edge transition in this system.^[^ ^11^ ^]^ From the above partial density of states analysis, it can be seen that Cl 3p states coupled with Ag 4d states in VBM of Cs_2_AgInCl_6_:Yb^3+^, which thus weakened the coupling of Cl and Yb and may be adverse to the Cl^−^‐Yb^3+^ charge transfer process in [YbCl_6_]^3−^ octahedron. By contrast, VBM of Cs_2_NaInCl_6_:Yb^3+^ was mainly composed of Cl 3p states without the contributions from Na, benefiting the coupling of Cl^−^ and Yb^3+^ and favoring the Cl^−^‐Yb^3+^ charge transfer process.

**Figure 4:**
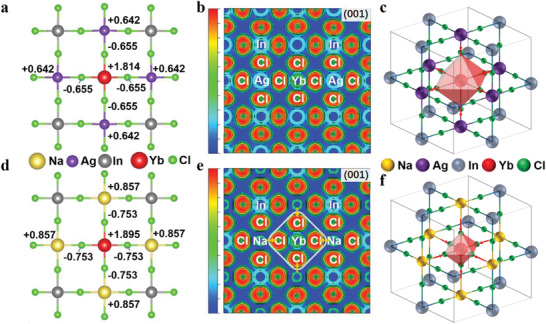
a) Bader charge analysis and b) ELF of Cs_2_AgInCl_6_:Yb^3+^. c) Schematic diagram of the structure of Cs_2_AgInCl_6_:Yb^3+^. d) Bader charge analysis and e) ELF of Cs_2_NaInCl_6_:Yb^3+^. f) Schematic diagram of the structure of Cs_2_NaInCl_6_:Yb^3+^.

The different electronic structures of Cs_2_NaInCl_6_:Yb^3+^ and Cs_2_AgInCl_6_:Yb^3+^ DPs were further verified by Bader charge analysis. In Cs_2_AgInCl_6_:Yb^3+^, Ag^+^ and Cl^−^ around Yb^3+^ had charge of +0.642 and −0.655, respectively. Besides, [YbCl_6_]^3−^ octahedron had a charge of −2.116, which confirmed that the electron of Cl^−^ ion was delocalized toward Ag^+^ due to the high covalency of the Ag—Cl bond (**Figure** **4**a).^[^ ^12^ ^]^ As such, the 3d orbit of Ag^+^ may catch electrons from Cl^−^, which thus impeded the charge transfer from Cl^−^ to Yb^3+^, as revealed by the electron localization function (ELF) analysis (Figure 4b,c).^[^ ^13^ ^]^ By contrast, Na^+^ ion in the Cs_2_NaInCl_6_:Yb^3+^ almost ionized completely with a charge of +0.857 and neighboring Cl^−^ with a charge of −0.753 (Figure 4a). Meanwhile, [YbCl_6_]^3−^ octahedron had a charge of −2.623, indicating that the electron may localize in the [YbCl_6_]^3−^ octahedron. Moreover, it was determined that the ELF between Na^+^ and Cl^−^ was almost zero due to the ionic bond characteristic (Figure 4e). Such weak interaction between Na and Cl in Cs_2_NaInCl_6_:Yb^3+^ may greatly promote the Cl^−^‐Yb^3+^ charge transfer process (Figure 4f).

**Figure 5:**
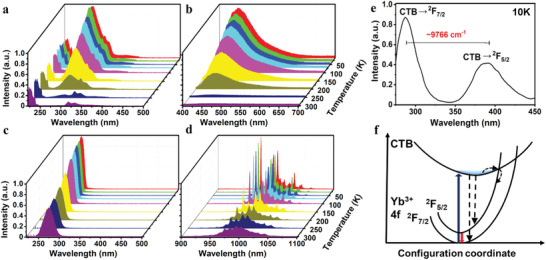
Temperature‐dependent a) excitation spectra (λ_em_ = 450 nm) and b) emission spectra (λ_ex_ = 290 nm) of Cs_2_NaInCl_6_. Temperature‐dependent c) excitation spectra (λ_em_ = 994 nm) and d) emission spectra (λ_ex_ = 273 nm) of Cs_2_NaInCl_6_:6.9% Yb^3+^. e) PL emission spectra of Cs_2_NaInCl_6_:6.9% Yb^3+^ at 10 K (λ_ex_ = 273 nm). f) Schematic illustration of the electronic transitions of Yb^3+^ in Cs_2_NaInCl_6_.

Furthermore, we carried out temperature‐dependent steady‐state and transient PL spectroscopic measurements to gain deep insights into the excited‐state dynamics of Yb^3+^ in Cs_2_NaInCl_6_. For pure Cs_2_NaInCl_6_, blue STE emission located at ≈450 nm with the FWHM of ≈75 nm was observed with temperatures below 200 K (**Figure** **5**a,b). The integrated intensity of STE at 10 K was 26.3 times higher than that at 300 K. Accordingly, the activation energy was determined to be 76 meV (Figure S6, Supporting Information), indicating excellent thermal stability of Cs_2_NaInCl_6_.^[^ ^14^ ^]^ The excitation spectra of STE peaking at ≈290 nm for Cs_2_NaInCl_6_ were associated with the bandgap absorption. Nevertheless, the excitation spectra of Yb^3+^ exhibited sharp peaks ranging from 265 to 273 nm for Cs_2_NaInCl_6_:Yb^3+^ (Figure 5c), which was distinct from the excitation spectra of pure Cs_2_NaInCl_6_, suggesting that they were originated from different processes. Upon excitation at 273 nm, a series of characteristic Yb^3+^ emission peaks were observed (Figure 5d). Besides, several vibronic peaks appeared at temperatures below 200 K, which were attributed to the vibrational modes of [YbCl_6_]^3−^ (Figure S7, Supporting Information).^[^ ^15^ ^]^ The PL lifetime of ^2^F_5/2_ of Yb^3+^ decreased from 8.17 ms at 10 K to 4.54 ms at 300 K due to the thermal quenching at high temperatures (Figure S6, Supporting Information).

Particularly, upon excitation at 273 nm at 10 K, two peaks with an energy gap of ≈9766 cm^−1^ were observed for Cs_2_NaInCl_6_:Yb^3+^, which agreed well with the energy gap between ^2^F_5/2_ and ^2^F_7/2_ of Yb^3+^ (Figure 5e). These two peaks can be attributed to the transitions from CTB to ^2^F_7/2_ (Yb^3+^) and ^2^F_5/2_ (Yb^3+^), respectively.^[^ ^10d^ ^]^ Such a result explicitly validated the existence of Cl^−^‐Yb^3+^ CTB.^[^ ^16^ ^]^ Thus, the energy transfer process of Yb^3+^ in Cs_2_NaInCl_6_ was proposed in Figure 5f. Upon UV excitation at 273 nm, the Yb^3+^ ion is excited from the 4f ground state (^2^F_7/2_) to the Cl^−^‐Yb^3+^ CTB, followed by a fast relaxation process to the 4f excited state (^2^F_5/2_) through thermal activation. Then, intense NIR emission of Yb^3+^ at 994 nm can be detected due to the radiative transition from ^2^F_5/2_ to ^2^F_7/2_.

**Figure 6:**
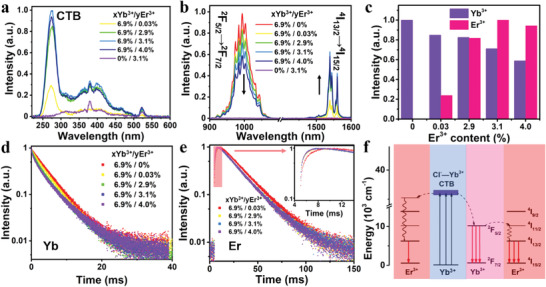
a) Excitation spectra (λ_em_ = 1540 nm) and b) emission spectra of Cs_2_NaInCl_6_ (λ_ex_ = 273 nm) doped with different contents of Yb^3+^ and Er^3+^. c) Integrated intensity of Yb^3+^ emission (purple) and Er^3+^ emission (pink) in Cs_2_NaInCl_6_:6.9%Yb^3+^/Er^3+^ with different contents of Er^3+^. d) PL decays of Yb^3+^ in Cs_2_NaInCl_6_:Yb^3+^/Er^3+^ with different contents Yb^3+^ and Er^3+^ by monitoring the emission at 994 nm. e) PL decays of Er^3+^ in Cs_2_NaInCl_6_:Yb^3+^/Er^3+^ with different contents Yb^3+^ and Er^3+^ by monitoring the emission at 1540 nm. The initial fast rise portion was enlarged in the inset. f) Schematic diagram of energy transfer process in Cs_2_NaInCl_6_ with simplified energy levels of Yb^3+^ and Er^3+^.

Besides Yb^3+^, another Ln^3+^ dopant, Er^3+^, was employed to produce NIR emissions (Table S5, Supporting Information). **Figure** **6**a shows the PL excitation spectra of Er^3+^ singly doped and Yb^3+^/Er^3+^ co‐doped Cs_2_NaInCl_6_ DPs. By monitoring the Er^3+^ emission at 1540 nm, the excitation peaks at 380 and 520 nm were detected for Cs_2_NaInCl_6_:Er^3+^ DPs, which belonged to ^4^I_15/2_ → ^4^G_11/2_ and ^4^I_15/2_ → ^2^H_11/2_ transitions of Er^3+^, respectively. For Cs_2_NaInCl_6_:Yb^3+^/Er^3+^ DPs, a strong peak at 273 nm corresponding to the Cl^−^‐Yb^3+^ CTB excitation appeared beside the above‐mentioned excitation peaks of Er^3+^. Upon excitation at 273 nm, Cs_2_NaInCl_6_:Yb^3+^/Er^3+^ DPs showed strong NIR emission peaking at 994 and 1540 nm corresponding to the ^2^F_5/2_ → ^2^F_7/2_ transition of Yb^3+^ and ^4^I_13/2_ → ^4^I_15/2_ of Er^3+^, respectively (Figure 6b). Note that the optimal integrated NIR intensity of Cs_2_NaInCl_6_:Yb^3+^/Er^3+^ DPs was 1510.2 times higher than that of Cs_2_NaInCl_6_:Er^3+^ counterparts (Figure 6b,c). The highest NIR PLQY of Cs_2_NaInCl_6_:Yb^3+^/Er^3+^ DPs was determined to be 7.9% (Table S4, Supporting Information).

Moreover, with the increase of Er^3+^ concentration in Cs_2_NaInCl_6_:6.9%Yb^3+^/xEr^3+^ DPs, it was discovered that the integrated intensity of Er^3+^ emission continuously increased while the Yb^3+^ emission steadily decreased, indicative of the energy transfer from Yb^3+^ to Er^3+^. Meanwhile, the PL lifetime of Yb^3+^ in Cs_2_NaInCl_6_:6.9%Yb^3+^/xEr^3+^ DPs decreased from 4.29 to 3.06 ms with the content of Er^3+^ increasing from 0.03% to 4.0%, which also verified the enhanced energy transfer from Yb^3+^ to Er^3+^ (Figure 6d). Furthermore, a decreased rising edge from 4.71 to 2.14 ms can be observed from the PL decays of Er^3+^ by monitoring the emission at 1540 nm, revealing the faster electron population process with increasing content of Er^3+^ (Figure 6e). The energy transfer efficiency (*η*_ET_) can be calculated as^[^ ^17^ ^]^

(1) $$\eta_{\mathrm{ET}}=1-\frac{\tau_{s}}{\tau_{0}}$$

where *τ*_0_ and *τ*_s_ display the Yb^3+^ lifetime (monitored at 994 nm) in the absence and presence of Er^3+^, respectively. Based on effective lifetime changes of different content Er^3+^‐doped Cs_2_NaInCl_6_:6.9%Yb^3+^/xEr^3+^ DPs, *η*_ET_ were calculated to be 11.3%, 23.4%, 29.5%, and 29.7% with the Er^3+^ content of 0.03%, 2.9%, 3.1%, and 4.0%, respectively. Thus, the energy transfer mechanism in Cs_2_NaInCl_6_:Yb^3+^/Er^3+^ is illustrated in Figure 6f. Upon excitation to the Cl^−^‐Yb^3+^ CTB, the excitation energy is transferred to the ^2^F_5/2_ (Yb^3+^) level through a fast nonradiative relaxation process, followed by the radiative transition of Yb^3+^ at 994 nm and energy transfer to the well‐matched ^4^I_11/2_ level of Er^3+^. Through the nonradiative relaxation from ^4^I_11/2_ to ^4^I_13/2_, the NIR emission at 1540 nm can be produced due to the ^4^I_13/2_ → ^4^I_15/2_ transition of Er^3+^.

## 3 Conclusion

In summary, we have unveiled the different local electronic structures of Ln^3+^ ions‐doped Cs_2_NaInCl_6_ DPs. Accordingly, a novel strategy for achieving efficient NIR luminescence of Ln^3+^ in Cs_2_NaInCl_6_ DPs was proposed, resulting in anNIR PLQY up to 39.4% of Yb^3+^ by virtue of the Cl^−^‐Yb^3+^ charge transfer sensitization. Through systematically investigating the PL excitation and emission spectra of Cs_2_AgInCl_6_:Yb^3+^ and Cs_2_NaInCl_6_:Yb^3+^, we revealed the superior sensitization paths of NIR emission of Yb^3+^ in Cs_2_NaInCl_6_ relative to that in Cs_2_AgInCl_6_. Notably, the Cs_2_NaInCl_6_:Yb^3+^ exhibited 142.2 times higher NIR PL intensity than the Cs_2_AgInCl_6_:Yb^3+^ counterparts. Temperature‐dependent PL excitation and emission spectra verified that the proposed Cl^−^‐Yb^3+^ charge transfer sensitization mechanism benefited from the localized electrons of [YbCl_6_]^3−^ octahedron in Cs_2_NaInCl_6_:Yb^3+^, which was also confirmed by the theoretical analysis. Furthermore, efficient NIR luminescence from Er^3+^ with PLQY of 7.9% was also achieved in Yb^3+^/Er^3+^ co‐doped Cs_2_NaInCl_6_ DPs due to the energy transfer from the Cl^−^‐Yb^3+^ CTB to Er^3+^. These findings provide a universal approach for the development of highly efficient Ln^3+^‐doped NIR luminescent halide DPs, which might pave a new way to manipulate the optical properties of Ln^3+^‐doped DPs toward versatile applications.

## Conflict of Interest

The authors declare no conflict of interest.

## Supporting information

Supporting Information

## Data Availability

The data that support the findings of this study are available from the corresponding author upon reasonable request.
