# Supplementary material for: Unveiling Local Electronic Structure of Lanthanide‐Doped Cs2NaInCl6 Double Perovskites for Realizing Efficient Near‐Infrared Luminescence
Source: Adv Sci (Weinh). 2022 Sep 30;9(32):2203735. doi: 10.1002/advs.202203735 (PMC9661838; doi:10.1002/advs.202203735)
Supplement: Supplementary file 1 — Supporting Information [file ADVS-9-2203735-s001.pdf]

ADVANCED SCIENCE
Open Access

Supporting Information

for *Adv. Sci.*, DOI 10.1002/advs.202203735

Unveiling Local Electronic Structure of Lanthanide-Doped $Cs_2NaInCl_6$ Double Perovskites for Realizing Efficient Near-Infrared Luminescence

Siyuan Han, Datao Tu, Zhi Xie, Yunqin Zhang, Jiayao Li, Yifan Pei, Jin Xu, Zhongliang Gong and Xueyuan Chen**

ADVANCED SCIENCE
Open Access

Supporting Information

for *Adv. Sci.*, DOI 10.1002/advs.202203735

Unveiling Local Electronic Structure of Lanthanide-Doped $Cs_2NaInCl_6$ Double Perovskites for Realizing Efficient Near-Infrared Luminescence

Siyuan Han, Datao Tu, Zhi Xie, Yunqin Zhang, Jiayao Li, Yifan Pei, Jin Xu, Zhongliang Gong and Xueyuan Chen**

Supporting Information
©Wiley-VCH 2021
69469 Weinheim, Germany

Unveiling local electronic structure of lanthanide-doped $Cs_2NaInCl_6$ double perovskites for realizing efficient near-infrared luminescence

Siyuan Han, Datao Tu, Zhi Xie, Yunqin Zhang, Jiayao Li, Yifan Pei, Jin Xu, Zhongliang Gong, and Xueyuan Chen**

SUPPORTING INFORMATION

Table of Contents

1. Experimental Procedures....................S2

2. Supporting Tables....................S3-S4

3. Supporting Figures....................S5-S10

4. References....................S11

5. Author Contributions....................S12

Experimental Procedures

Chemicals and materials: Caesium chloride (CsCl, 99.999%) and isopropyl alcohol (99.9%) were purchased from Aladdin. Sodium chloride (NaCl, 99.5%) and hydrochloric acid (HCl, 37%) were purchased from Sinopharm Chemical Reagent Co. (Shanghai, China). Indium acetate ($InAc_3$, 99.99%) and silver chloride (AgCl, 99.9%) were purchased from Alfa Aesar. $Yb(Ac)_3$•xH_2O (99.9%) was purchased from Sigma-Aldrich. All the chemical reagents were of analytical grade and used as received without further purification unless otherwise noted. Distilled water was used throughout the experiments.

Synthesis of $Cs_2Na_xAg_{1-x}InCl_6$:Yb^{3+} Crystals: High-quality lanthanide-doped $Cs_2Na_xAg_{1-x}InCl_6$ double perovskites (DPs) were synthesized via a facile one-pot hydrothermal synthesis method. In a typical synthesis, 1.6 mmol of CsCl, 0.8*x mmol of NaCl, 0.8*(1-x) mmol of AgCl, 0.8 mmol of $InAc_3$ and 1.2 mmol of $YbAc_3$ were dissolved in 8.0 mL of HCl (10 M) solution in a 20-mL Teflon autoclave. Then the solution was heated at 180 °C for 12 h in a stainless-steel autoclave. The solution was then steadily cooled down to 30 °C at a speed of 3 °C h^{-1}. Then the crystals were filtered out, washed three times with isopropyl alcohol to remove the solvent from the crystal surface and dried in an oven at 60 °C. Finally, the crystals were ground into powder as a phosphor.

Synthesis of $Cs_2NaInCl_6$:Ln^{3+} Crystals: The synthesis method of $Cs_2NaInCl_6$:xLn^{3+} is similar with the above synthesis process. $Cs_2NaInCl_6$:xLn^{3+} were obtained with different concentration of precursors of $YbAc_3$ (x = 0.4, 0.8, 1.2, 1.6 mmol) or $ErAc_3$ (x = 0.24, 0.48, 0.72, 0.96 mmol) and 0.8 mmol of NaCl to adjust the contents of lanthanide dopants in $Cs_2NaInCl_6$.

Synthesis of $Cs_2AgInCl_6$:Ln^{3+} Crystals: The synthesis method of $Cs_2AgInCl_6$:xLn^{3+} is similar to the above synthesis process of $Cs_2NaInCl_6$:xLn^{3+}, wherein the only difference is that 0.8 mmol of AgCl was employed instead of NaCl.

Photoluminescence (PL) quantum yield (QY) measurement: A barium sulfate-coated integrating sphere (150 mm in diameter, Edinburgh) was employed as the sample chamber that was mounted by a fiber optic spectrometer (QE65pro, Ocean Optics) with the entry and output port of the sphere located in 90° geometry from each other in the plane of the spectrometer. The Near-Infrared (NIR) emission in the spectral range of 900-1100 nm for Yb^{3+} and 1500-1600 nm for Er^{3+} were integrated for the QY determination. All the spectral data collected were corrected for the spectral response of both the spectrometer and the integrating sphere. We calculated the absolute PLQY based on the following equation:

$$PLQY=\frac{N_e}{N_a}=\frac{L_s}{E_r-E_s}$$

where N_e and N_a are the photons emitted and absorbed, respectively; L_s is the emission intensity, E_r and E_s are the intensities of the excitation light in the presence of the $BaSO_4$ (reference) and Yb^{3+} (Er^{3+}) doped $Cs_2NaInCl_6$ (sample) DPs, respectively. All the PLQYs for each sample were measured independently at least three times under identical conditions to yield the average value.

Characterization: Powder X-ray diffraction (XRD) patterns of the samples were collected with an X-ray diffractometer (MiniFlex 600, Rigaku) with Cu $K_{\alpha 1}$ radiation (λ = 0.154187 nm), operating at 40 kV and 40 mA. Inductively coupled plasma atomic emission spectroscopy (ICP-AES) measurements were performed at inductively coupled plasma atomic emission spectroscopy spectrometry (Ultima2, HORIBA Jobin Yvon). The diffuse reflectance spectra were recorded on a UV–vis–NIR spectrophotometer (Lambda950). X-ray photoelectron spectroscopy (XPS) measurements were performed on an ESCALAB 250Xi X-ray Photoelectron Spectroscopy using a monochromatic Al Kα source (15 kV, 20 mA). The PL emission and PL excitation (PLE) spectra and PL decay curves were recorded on an Edinburgh FLS980 spectrofluorometer equipped with a continuous xenon lamp (450 W), a pulsed flash lamp and a 375 nm picosecond pulsed laser. Effective PL lifetimes (τ_{eff}) were calculated by:

$$\tau_{eff}=\frac{1}{I_{\max}}\int_0^\infty I(t)dt$$

where $I(t)$ denotes the PL intensity as a function of time t, and I_{max} is the maximum PL intensity.

Density functional theory (DFT) Calculation Details: The density functional theory (DFT) calculation was performed using the Vienna Ab-Initio Simulation Package. [1, 2] The electron-ion interaction was described by projector augmentedwave (PAW) pseudopotentials. For the exchange and correlation functionals, we use the Perdew-Burke-Ernzerhof (PBE) version of the generalized gradient approximation (GGA) exchange-correlation. [3] The energy cutoff of 500 eV was used for the wave functions expansion. The energy and force converged to 1.0 × 10-5 eV $atom^{-1}$ and 0.05 eV·$Å^{-1}$. The Brillouin zone integration was sampled with 2 × 2 × 2 k-grid mesh. The Heyd-Scuseria-Ernzerhof (HSE06) calculations were employed to obtain accurate bandgap. [4]

Table S1. ICP-AES results of Yb^{3+}-doped $Cs_2AgInCl_6$ crystals with different Yb^{3+} feeding ratios. The feeding concentrations of Yb^{3+} were from 50% to 200%, while the actual Yb^{3+} concentrations in the crystal lattice were only from 1% to 15.5%.

Yb feeding (%)	In (%)	Yb (%)
50	99.0	1.0
100	98.3	1.7
150	94.2	5.8
200	84.5	15.5

Table S2. ICP-AES results of Yb^{3+}-doped $Cs_2NaInCl_6$ crystals with different Yb^{3+} feeding ratios. The feeding concentrations of Yb^{3+} were from 50% to 200%, while the actual Yb^{3+} concentrations in the crystal lattice were only from 0.4% to 8.7%.

Yb feeding (%)	In (%)	Yb (%)
50	99.6	0.4
100	99.1	0.9
150	93.1	6.9
200	91.3	8.7

Table S3. ICP-AES results of Yb^{3+}-doped $Cs_2NaInCl_6$ crystals with different synthesis conditions. The feeding concentrations of Yb^{3+} were 150%, while the actual Yb^{3+} concentrations in the crystal lattice remained essentially unchanged with the result of our work.

Synthesis conditions		Yb (%)
Reaction temperature (°C)	Cooling time (h)	
180	48	6.9 (this work)
220	48	6.8
180	72	7.2

Table S4. Comparison of the PL excitation (λ_{ex}), emission (λ_{em}) wavelengths and PLQYs of $Cs_2NaInCl_6$:Ln^{3+} with those of the Ln^{3+}-doped NIR luminescent lead-free metal halides recently reported. The highest PLQY of Yb^{3+} in $Cs_2NaInCl_6$:Yb^{3+} reaches 39.4% via charge transfer band (CTB) sensitization, which is a high PLQY among the lead-free halide DPs.

Compositions	Samples	λ_{ex} / nm	λ_{em} / nm	Sensitization	PLQY / %	Ref
$Cs_2AgInCl_6$:Yb^{3+}	NCs	300	996	STE	3.6	[5]
$Cs_2AgBiBr_6$:Yb^{3+}	Film	430	1000	STE	28	[6]
$Cs_3Bi_2Br_9$:Yb^{3+}	Film	360	993	STE	14.5	[7]
$Cs_2Na_{0.2}Ag_{0.8}InCl_6$: Yb^{3+}	Crystals	365	996	STE	46	[8]
**$Cs_2NaInCl_6$:Yb^{3+}**	**MCs**	**273**	**994**	**CTB**	**39.4**	**This work**
Cs_2ZrCl_6: Te^{4+}/Yb^{3+}	MCs	392	1002	Te^{4+}	0.2	[9]
$Cs_2AgInCl_6$:Er^{3+}	NCs	300	1537	STE	0.05	[5]
$Cs_2NaSbCl_6$:Er^{3+}	NCs	335	1543	STE	0.26	[10]
$Cs_2NaBiCl_6$:Mn^{2+}/Er^{3+}	MCs	360	1540	STE	14.2	[11]
$Cs_2Ag_{0.6}Na_{0.4}InCl_6$:$Bi^{3+}$/$Er^{3+}$	MCs	365	1540	STE	38.3	[12]
**$Cs_2NaInCl_6$:Yb^{3+}/Er^{3+}**	**MCs**	**273**	**1540**	**CTB**	**7.9**	**This work**
Cs_2ZrCl_6: Te^{4+}/Er^{3+}	MCs	392	1539	Te^{4+}	6.1	[9]

Table S5. ICP-AES results of Er^{3+}-doped $Cs_2NaInCl_6$ crystals with different Er^{3+} feeding ratios. The feeding concentrations of Er^{3+} were from 30% to 120%, while the actual Er^{3+} concentrations in the crystal lattice were only from 0.03% to 4.0%.

Er feeding (%)	In (%)	Yb (%)	Er (%)
30	90.07	6.9	0.03
60	90.2	6.9	2.9
90	90.0	6.9	3.1
120	89.1	6.9	4.0

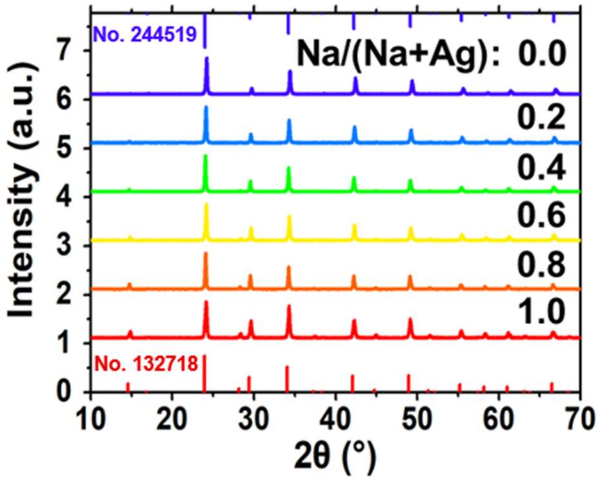

Figure S1. XRD patterns of $Cs_2Na_xAg_{1-x}InCl_6$ crystals with different Na/(Na+Ag) ratios. The crystals can be well indexed into cubic $Cs_2AgInCl_6$ (ICSD No. 244519) and $Cs_2NaInCl_6$ (ICSD No. 132718) without any impurities.

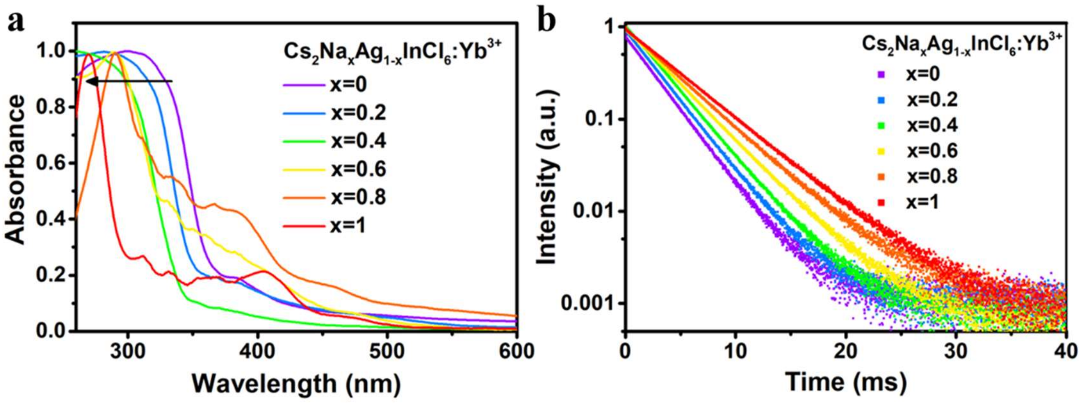

Figure S2. (a) Diffuse reflectance spectra of $Cs_2Na_xAg_{1-x}InCl_6:Yb^{3+}$ with different Na/(Na+Ag) ratio. The absorbance band of $Cs_2Na_xAg_{1-x}InCl_6:Yb^{3+}$ crystals located in the UV regions. The band edges shifted from 355 nm to 283 nm as the Na/(Na+Ag) ratio increased from 0 to 1. (b) PL decay of Yb^{3+} in $Cs_2Na_xAg_{1-x}InCl_6:Yb^{3+}$ by monitoring the emission at 994 nm with different Na/(Na+Ag) ratios. The lifetimes of Yb^{3+} were determined to be 2.72 ms, 2.84 ms, 3.11 ms, 3.84 ms, 4.04 ms, 4.52 ms with increasing the Na/(Na+Ag) ratios from 0 to 1.

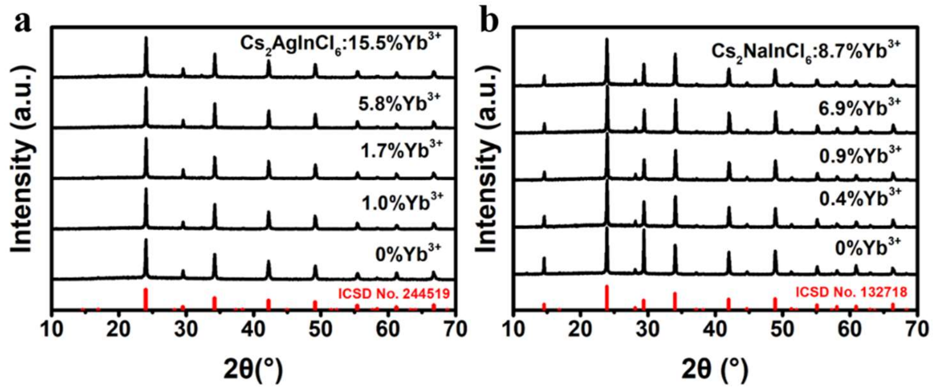

Figure S3. (a) XRD patterns of $Cs_2AgInCl_6$:Yb^{3+} with different concentrations of Yb^{3+}. All these $Cs_2AgInCl_6$:Yb^{3+} samples can be well indexed into cubic $Cs_2AgInCl_6$ (ICSD No. 244519). (b) XRD patterns of $Cs_2NaInCl_6$:Yb^{3+} with different concentrations of Yb^{3+}. All these $Cs_2NaInCl_6$:Yb^{3+} can be well indexed into cubic $Cs_2NaInCl_6$ (ICSD No. 132718).

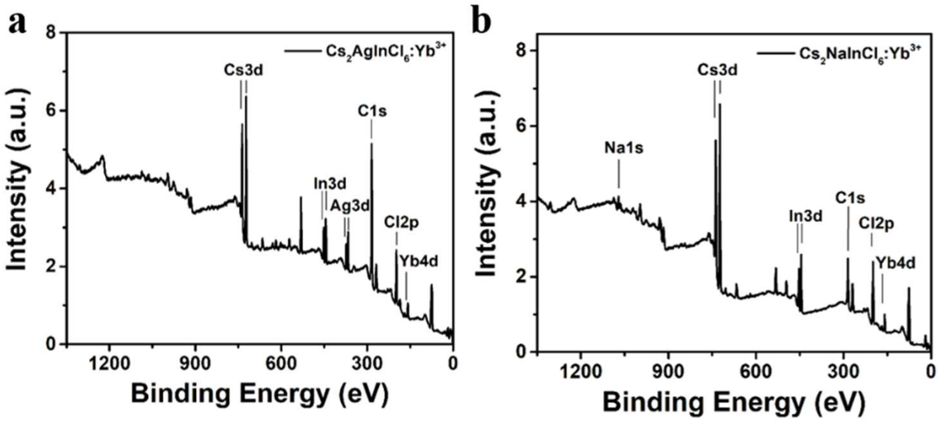

Figure S4. (a) XPS analyses of $Cs_2AgInCl_6$:Yb^{3+} and (b) $Cs_2NaInCl_6$:Yb^{3+}. Both spectra were corrected by C-C at 284.8eV. The peaks centered at 738.2 and 724.3 eV belonged to Cs, 1071.6 eV belonged to Na, 373.6 and 367.7 eV belonged to Ag, 445.7 and 453.6 eV belonged to In, 199.1 and 200.6 eV belonged to Cl, 185.7 eV belonged to Yb. XPS analyses revealed the existence of Yb^{3+} ions in the as-prepared DPs.

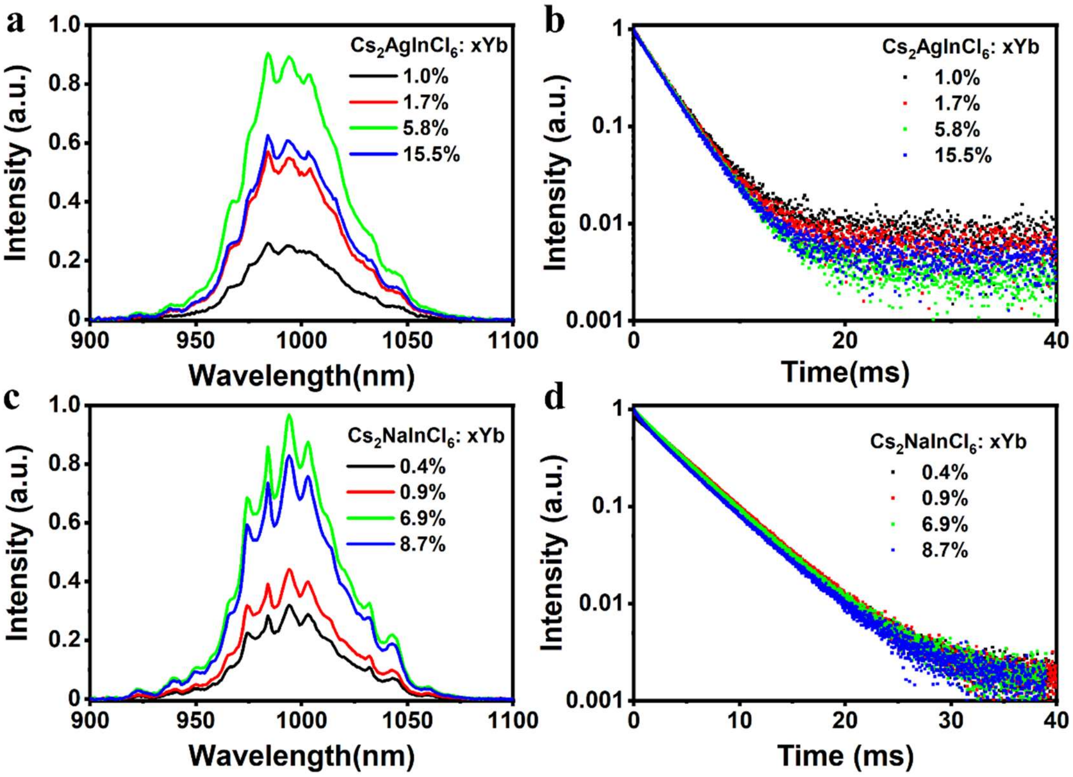

Figure S5. (a) PL spectra of $Cs_2AgInCl_6$:Yb^{3+} with different content of Yb^{3+}. Upon excitation at 365 nm, these $Cs_2AgInCl_6$:Yb^{3+} samples exhibited NIR PL with characteristic peaks at 994 nm, corresponding to the $^2F_{5/2} \rightarrow {}^2F_{7/2}$ transition of Yb^{3+}. (b) PL decay of Yb^{3+} in $Cs_2AgInCl_6$:Yb^{3+} by monitoring the emission at 994 nm. PL decays revealed decreased PL lifetime from 2.76 ms to 2.54 ms with the concentration of Yb^{3+} from 1.0% to 15.5%. (c) PL spectra of $Cs_2NaInCl_6$ with different content of Yb^{3+}. Upon excitation at 273 nm, the characteristic emission of Yb^{3+} was observed. The NIR luminescence intensity of $Cs_2NaInCl_6$:Yb^{3+} was ~142.2 times higher than that of $Cs_2AgInCl_6$:Yb^{3+} with the optimal doping concentration (6.9%). (d) PL decay of 994 nm with different Yb^{3+} concentrations. The PL lifetime of Yb^{3+} in $Cs_2NaInCl_6$:Yb^{3+} was determined to decrease from 4.54 ms to 4.11 ms with the concentration of Yb^{3+} increased from 0.4 mol% to 8.7 mol%.

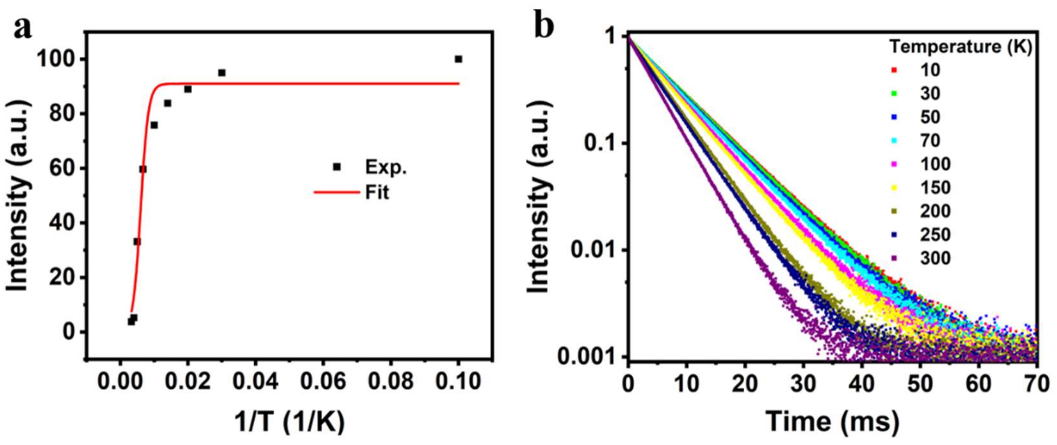

Figure S6. (a) Integrated PL intensity of pure $Cs_2NaInCl_6$ determined from the temperature-dependent PL spectra. The activation energy was determined to be 76 meV, indicating excellent thermal stability of $Cs_2NaInCl_6$. (b) Temperature-dependent PL decays of $Cs_2NaInCl_6$:6.9% Yb^{3+} by monitoring the Yb^{3+} emission at 994 nm. The PL lifetime of $^2F_{5/2}$ of Yb^{3+} decreased from 8.17 ms at 10 K to 4.54 ms at 300 K due to the thermal quenching at high temperatures.

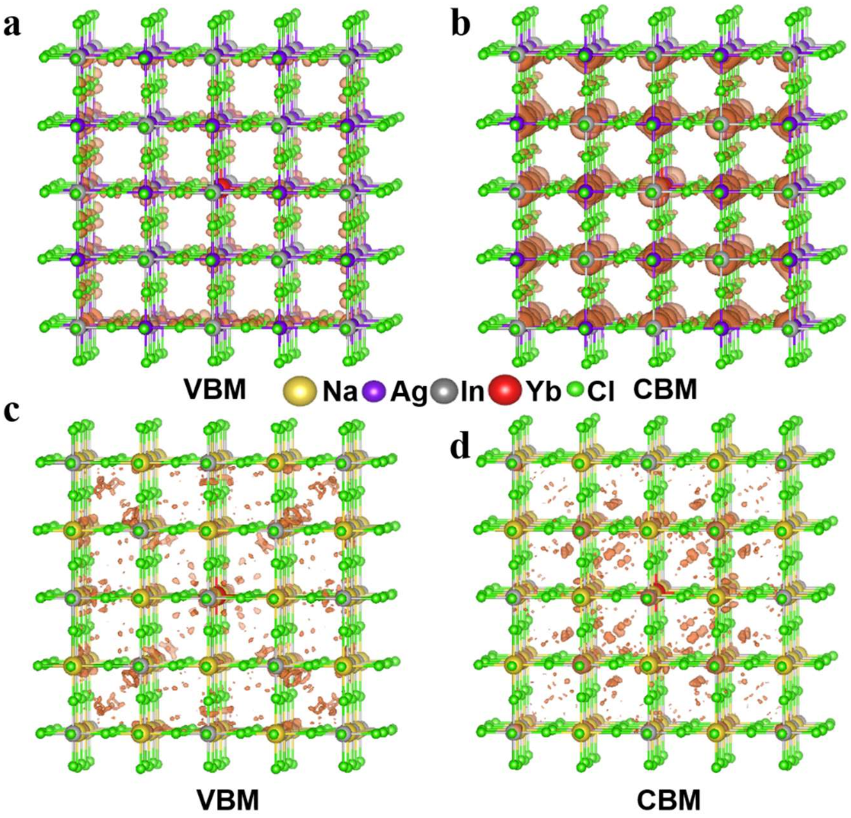

Figure S7. (a) Orbital distribution profiles of valence band maximum (VBM) in $Cs_2AgInCl_6$:Yb^{3+}. (b) Orbital distribution profiles of conduction band minimum (CBM) in $Cs_2AgInCl_6$:Yb^{3+}. (c) Orbital distribution profiles of VBM in $Cs_2NaInCl_6$:Yb^{3+}. (d) Orbital distribution profiles of CBM in $Cs_2NaInCl_6$:Yb^{3+}. Orbital distribution profiles of $Cs_2AgInCl_6$:Yb^{3+} showed that VBM was composed of a mixed configuration of Ag 4d and Cl 3p states, and CBM mainly consisted of In 5s states with minor contributions from Ag 4d and Cl 3p states. Such configuration benefited the formation of STE, which resulted from the Jahn-Teller distortion of the connected $[AgCl_6]^{5-}$-$[InCl_6]^{3-}$ octahedron. For $Cs_2NaInCl_6$:Yb^{3+}, VBM and CBM were essentially composed of Cl 3p states and In 5s states, respectively, which revealed that the orbitals were distributed over the whole supercell with little spatial overlap. Such poor spatial overlap led to the extremely weak edge-to-edge transition in this system.

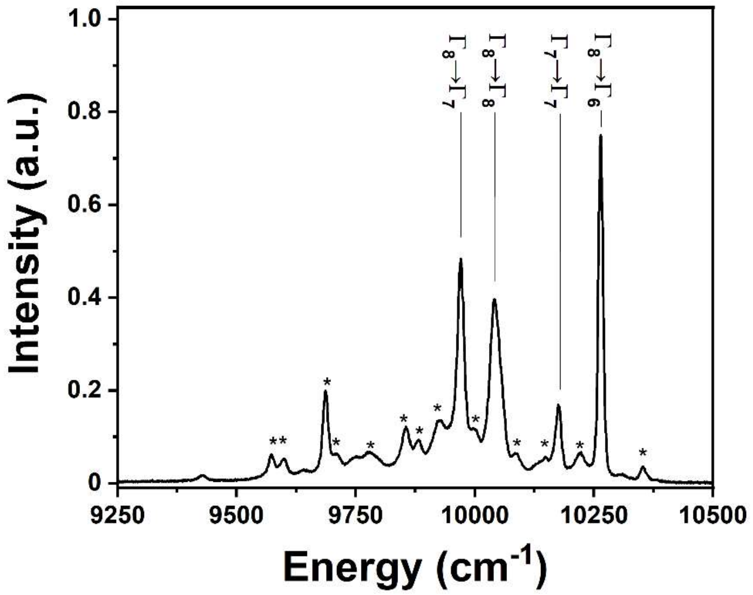

Figure S8. Vibronic PL spectra of $Cs_2NaInCl_6$:Yb^{3+} at 10 K. The intense $^2F_{5/2}$ $\Gamma_8 \rightarrow \Gamma_6$, Γ_7, Γ_8 $^2F_{7/2}$, and $^2F_{5/2}$ $\Gamma_7 \rightarrow \Gamma_7$ $^2F_{7/2}$ transitions of Yb^{3+} can be observed. Each transition shows rich vibronic emission (peaks with '*') deriving from vibrational modes of $[YbCl_6]^{3-}$. [13]

References

[1] G. Kresse, J. Furthmüller, Efficient iterative schemes for ab initio total-energy calculations using a plane-wave basis set, Phys. Rev. B, **1996** *54* 11169.

[2] G. Kresse, D. Joubert, From ultrasoft pseudopotentials to the projector augmented-wave method, Phys. Rev. B, **1999** *59* 1758.

[3] J.P. Perdew, K. Burke, M. Ernzerhof, Generalized gradient approximation made simple, Phys. Rev. Lett., **1996** *77* 3865.

[4] M. Marsman, J. Paier, A. Stroppa, G. Kresse, Hybrid functionals applied to extended systems, J. Phys.: Condens. Matter, **2008** *20* 064201.

[5] W. Lee, S. Hong, S. Kim, *J. Phys. Chem. C* **2019**, *123*, 2665.

[6] F. Schmitz, K. P. Guo, J. Horn, R. Sorrentino, G. Conforto, F. Lamberti, R. Brescia, F. Drago, M. Prato, Z. B. He, U. Giovanella, F. Cacialli, D. Schlettwein, D. Meggiolaro, T. Gatti, *J. Phys. Chem. Lett.* **2020**, *11*, 8893.

[7] M. N. Tran, I. J. Cleveland, G. A. Pustorino, E. S. Aydil, *J. Mater. Chem. A* **2021**, *9*, 13026.

[8] N. Q. Liu, W. Zheng, R. J. Sun, X. L. Li, X. Y. Xie, L. L. Wang, Y. H. Zhang, *Adv. Funct. Mater.* **2022**, *32*, 2110663.

[9] J. Sun, W. Zheng, P. Huang, M. Zhang, W. Zhang, Z. Deng, S. Yu, M. Jin, X. Chen, *Angew. Chem. Int. Ed.* **2022**, e202201993

[10] R. X. Wu, P. G. Han, D. Y. Zheng, J. F. Zhang, S. Q. Yang, Y. Zhao, X. Y. Miao, K. L. Han, *Laser Photon. Rev.* **2021**, *15*, 2100218.

[11] W. Gan, B.-M. Liu, L. Huang, S. Lou, J. Zhang, Z. Zhou, J. Wang, *Adv. Optical Mater.* **2022**, DOI: 10.1002/adom.202102851.

[12] J. Zhao, G. Pan, K. Liu, W. You, S. Jin, Y. Zhu, H. Gao, H. Zhang, Y. Mao, *J. Alloys Compd.* **2022**, *895*, 162601.

[13] a) L. X. Ning, P. A. Tanner, S. D. Xia, *Vib. Spectrosc* **2003**, *31*, 51; b) R. Acevedo, P. A. Tanner, T. Meruane, V. Poblete, *Phys. Rev. B* **1996**, *54*, 3976.

Author Contributions

Siyuan Han, Datao Tu and Xueyuan Chen conceived the project, wrote the paper, and were primarily responsible for the experiments. All authors contributed to the analysis of the manuscript.
